# Developing a Smoking Cessation Intervention for People With Severe Mental Illness Treated by Flexible Assertive Community Treatment Teams in the Netherlands: A Delphi Study

**DOI:** 10.3389/fpsyt.2022.866779

**Published:** 2022-07-06

**Authors:** Müge H. Küçükaksu, Trynke Hoekstra, Lola Jansen, Jentien Vermeulen, Marcel C. Adriaanse, Berno van Meijel

**Affiliations:** ^1^Department of Health Sciences and Amsterdam Public Health Research Institute, Vrije Universiteit Amsterdam, Amsterdam, Netherlands; ^2^Department of Psychiatry, Amsterdam University Medical Centre (UMC), University of Amsterdam, Amsterdam, Netherlands; ^3^Department of Psychiatry, Amsterdam University Medical Centre (UMC) and Amsterdam Public Health Research Institute, Amsterdam, Netherlands; ^4^Department of Health, Sports and Welfare, Inholland University of Applied Sciences, Amsterdam, Netherlands; ^5^Parnassia Psychiatric Institute, Parnassia Academy, The Hague, Netherlands

**Keywords:** tobacco addiction, outpatient psychiatric care, behavioural counselling, psychotic disorders, schizophrenia, bipolar disorder, pharmacotherapy for smoking cessation

## Abstract

**Background:**

There is still limited evidence on the effectiveness and implementation of smoking cessation interventions for people with severe mental illness (SMI) in Dutch outpatient psychiatric settings. The present study aimed to establish expert consensus on the core components and strategies to optimise practical implementation of a smoking cessation intervention for people treated by Flexible Assertive Community Treatment (FACT) teams in the Netherlands.

**Design:**

A modified Delphi method was applied to reach consensus on three core components (behavioural counselling, pharmacological treatment and peer support) of the intervention. The Delphi panel comprised five experts with different professional backgrounds. We proposed a first intervention concept. The panel critically examined the evolving concept in three iterative rounds of 90 min each. Responses were recorded, transcribed verbatim and thematically analysed.

**Results:**

Overall, results yielded that behavioural counselling should focus on preparation for smoking cessation, guidance, relapse prevention and normalisation. Pharmacological treatment consisting of nicotine replacement therapy (NRT), Varenicline or Bupropion, under supervision of a psychiatrist, was recommended. The panel agreed on integrating peer support as a regular part of the intervention, thus fostering emotional and practical support among patients. Treatment of a co-morbid cannabis use disorder needs to be integrated into the intervention if indicated. Regarding implementation, staff's motivation to support smoking cessation was considered essential. For each ambulatory team, two mental health care professionals will have a central role in delivering the intervention.

**Conclusions:**

This study provides insight into expert consensus on the core components of a smoking cessation intervention for people with SMI. The results of this study were used for the development of a comprehensive smoking cessation program.

## Introduction

Smoking is the leading factor associated with cardiovascular diseases, cancers and diseases of the respiratory system, causing nearly eight million deaths worldwide each year (1). People with severe mental illness (SMI), such as psychosis, bipolar disorder or severe depression, are affected more often by tobacco addiction, with the proportion of smokers among these patients being 2–3 times higher compared to the general population (2–4). They also have more difficulties with overcoming addiction, manifested in more quit attempts and relapses, thus widening health inequalities between the general and psychiatric population (5, 6). Additionally, the proportion of smokers in the general population showed an evident decline over the past decade, while this proportion among people with SMI did not show a decrease, but rather a stagnation (4).

There are several possible explanations underlying the high prevalence of smoking in SMI, which can be interconnected and include models of shared genetic, psychological, social and environmental risk factors. Research on the relationship between nicotine addiction and psychosis, for instance, showed some shared genetic liability (7, 8). In individuals with a pre-existing vulnerability for psychosis, cigarette smoking may lead to an earlier onset of psychosis compared to non-smokers (9). Another study showed a significant positive association between smoking and the frequency by which positive, negative and depressive symptoms are experienced, as well as an increase in positive symptoms in patients who started to smoke (10). These results suggest a potential bidirectional relationship between psychosis and smoking and further push forward the need for smoking interventions in this patient group. Shared social and environmental risk factors involve, amongst others, a lower socioeconomic status (SES). Low SES has been associated with higher smoking prevalence and social acceptance of smoking. At the same time, a low SES early in life increases the risk for psychiatric disorders, possibly due to a correlation with structural disadvantage, parental mental illness and childhood adversity (11). Taken together, these different notions are possible explanations for the very prevalent co-occurrence of smoking and SMI and describe mechanisms that cause and maintain tobacco addiction.

Another emerging concept is emotion dysregulation—a transdiagnostic factor in psychopathology, particularly for personality disorders and mood disorders—that is also associated with heavier smoking and more difficulties with quitting (12, 13). The idea of emotion dysregulation adds to the belief that cigarette smoking may have the potential to attenuate symptoms, such as depressive symptoms or cognitive problems (e.g., concentration and attention problems), and to counterbalance side effects of antipsychotic medication (e.g., increased appetite) (14). Although there is no clear evidence to support this self-medication hypothesis (10), its underlying beliefs have contributed to the social acceptance of smoking within mental health care (15). A couple of notions may have impeded the collective process of critically evaluating the association between smoking on the one hand, and psychiatric symptoms, somatic health and quality of life on the other hand: health care professionals' view that smoking may be helpful for people with SMI and therapeutic pessimism regarding both general treatment outcomes and opportunities for successfully quitting smoking.

In recent years, the Netherlands, among other countries, has introduced new policy measures to raise awareness regarding the negative impact of tobacco use, and a smoking ban in public areas, including mental health care institutions. As a result of these developments, there is a need for more evidence-based interventions for smoking cessation in mental health care settings (16, 17).

There is compelling evidence on the effectiveness of behavioural support and pharmacological treatment for smoking cessation among people with SMI. For behavioural support, the two most commonly used and researched therapeutic approaches to treat addiction are cognitive-behavioural therapy (CBT) and motivational interviewing (MI) (18). CBT for smoking cessation is comparably effective for people with and without SMI (19, 20). Moreover, studies comparing the effectiveness of CBT alone to CBT combined with pharmacotherapy showed that a combination of both is the most effective for smoking cessation (20–22). Regarding pharmacotherapy, a series of trials that examined the safety and effectiveness of Varenicline, Bupropion and nicotine replacement therapy (NRT) for people with SMI, showed overall positive results in favour of the use of these medications (23, 24). In addition to these therapeutic and medical interventions, peer support can add a source for social support and improve a person's social network—a decisive factor for smoking cessation (25). Peer support appears to be particularly relevant in the present population, in which persons often have small social networks.

A previous clinical trial on the treatment of tobacco addiction in psychiatric patients, showed that using a combination of these components was superior to care as usual (26, 27). Despite basic knowledge of the core components of a smoking cessation intervention for patients with SMI, there is a need for additional insights into the specific content of these components (following the most recent practical and scientific knowledge), on how to better tailor these to the needs of this population, and how to effectively implement them in Dutch mental health care.

Prior to a planned randomised controlled trial (RCT), which will evaluate the implementation and effectiveness of a smoking cessation program in ambulatory mental health care, we carried out a Delphi study to reach consensus on the specific content of such a program. Additional aims were to incorporate country- and time-specific characteristics of the mental health care settings (e.g., institutional restructuring following a new insurance policy and local measurements to prevent the spread of COVID-19) in which the program will be implemented (28). To the best of our knowledge, this is the first study that aims to identify and reach consensus about the structure and content of a smoking cessation intervention offered to people with severe mental illness in an outpatient clinical setting in the Netherlands.

## Methods

### Study Design

We conducted a modified three-round Delphi study with five experts on smoking cessation, with different expertise and backgrounds (29). In light of the ongoing COVID-19 pandemic, all rounds were held online *via* videoconferencing software Zoom.us between December 2020 and February 2021. Using Zoom for qualitative research is well-accepted and perceived as convenient by researchers and participants (30).

### Selection of Participants

We selected five experts aged between 31 and 64. Number of years of experience with treating tobacco addiction in people with SMI ranged from 3 to 10 years. Participants were recruited through the researchers' professional networks. Considering that mental health care nurses working in ambulatory mental health teams will be delivering the intervention, we included two clinical nurse specialists with ample clinical experience with smoking cessation among SMI patients. To ensure the incorporation of clients' perspectives we included an expert-by-experience. We also included a practising physician/researcher, with comprehensive clinical and research experience on smoking cessation and early psychosis. Finally, a senior project leader and consultant of tobacco regulation in mental health care in the Netherlands was included.

### Data Collection

The overall aim during all three rounds was to reach consensus about the composition of the three central components of the smoking cessation intervention, and strategies to optimise implementation in clinical practise. Participants were invited and informed through an electronic invitation letter. All rounds, with a duration of 90 min, were semi-structured and recorded for analysis. MK prepared the Delphi procedures and processed all responses. To compensate for two participants' absence during the group interviews on two occasions, individual interviews were conducted with three of the researchers (TH, MA and MK). BvM moderated the panel sessions while MA moderated the two individual sessions. Two weeks before the first round, participants received three documents for preparation:

1. An overview of the procedures of the Delphi study, as well as a description of what participation in the panel entails.
2. The smoking cessation intervention concept describing propositional components and elements of the intervention, including their rationale, theoretical background and context (31).
3. Nine open-ended questions to stimulate general feedback on the first concept version (see Supplementary Material *Interview Guide*).

**Figure 1:**
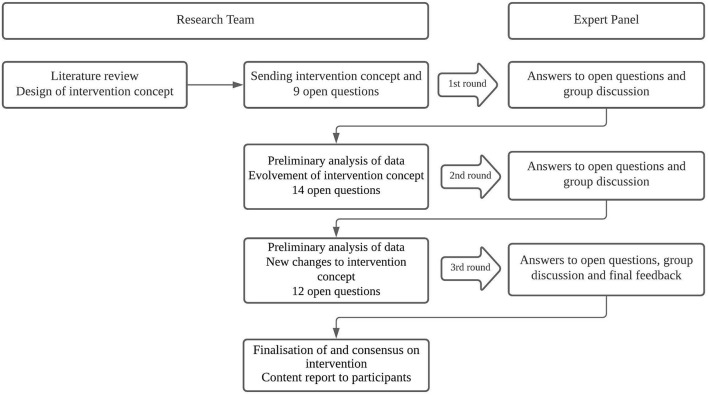
Flowchart of the three-round Delphi procedure.

Responses to the open-ended questions were received through e-mail from each participant before the first panel session. In summary, the structure of the three rounds was as follows (see also Figure 1):

*Round 1:* Each question (*n* = 9) and participants' responses were reviewed and discussed. The experts' contributions of the first round were then thematically summarised. The focus during this round was on the general structure of the intervention program.
*Round 2:* Participants received 14 new questions based on preliminary outcomes from round 1. Participants also received a new draught of the intervention concept based on the first round. Responses to the 14 open-ended questions were deliberated during round 2. The specific focus during this second round was on the use of e-cigarettes, strategies for relapse (prevention), and the involvement of peers and family members.
*Round 3:* Participants received an overview with preliminary conclusions drawn from the first two rounds and 12 final open questions. In this round, the discussion focused, among other things, on the ratio of individual and group behavioural support, concrete guidelines for pharmacological treatment and how to deal with comorbid cannabis use disorder.

Differences in opinions were regarded as opportunities to explore these discrepancies and find compromise for the intervention design and its implementation. After each round, the research team debriefed, and points of disagreement were the starting point for the next round. After the final round, the panel received a definitive version of the intervention. The panel reached eventual consensus through negotiation, taking into consideration the expected effectiveness of the component, the needs of clients, treatment possibilities of clinical staff and practical conditions for implementation in psychiatric institutions.

### Ethical Considerations

We obtained consent for video and audio recordings beforehand from all participants. The transcripts were pseudonymised. Monetary compensation of 1,100 euros for preparation and participation in all three rounds was offered, which participants received after the last round.

### Analysis

Thematic analysis was applied (32). Two authors (MK and LJ) transcribed all audio recordings verbatim. The authors familiarised themselves with the data by listening to the interviews. Subsequently, the transcripts were coded using MAXQDA. As there was already a predefined intervention concept, the three intervention components (1. Behavioural counselling based on CBT and MI techniques; 2. Pharmacological treatment; 3. Peer support) and aspects of practical implementation were defined as an initial framework before coding. Based on this framework, code words and themes were generated. New code words and themes, that emerged from the data, were added. Next, the research team reviewed the generated themes and discussed discrepancies if needed.

## Results

### Intervention Components

#### Behavioural Counselling

The initial phase of behavioural counselling prepares the patient for the actual quitting moment through psycho-education, assessment of motivation to quit and identification of individual support needs. Although individual counselling was regarded as therapeutically effective and should be actively suggested to patients, there was consensus among the experts to offer group sessions per default once a week. For reasons of limited staff capacity within clinical teams, individual consults are available upon demand. The panel also noted that group sessions could strengthen patients' social connectedness and that motivation to quit smoking was enhanced by mutual contacts within the group of patients. This aspect can be additionally reinforced by peer support meetings. At the same time, constraints of group sessions, such as cognitive overstimulation and concentration problems, should be taken into consideration by introducing sufficient breaks and facilitating new content with, for instance, visual material.

Further, all participants agreed to emphasise relapse prevention and normalisation of relapse as well as the differentiation between relapse and “slips.” While “slips” refer to a momentary give-in to craving (e.g., smoking one cigarette), relapse entails returning to a regular smoking pattern similar or identical to before quitting. Relapse and “slips” need to be addressed explicitly as common parts in overcoming addiction and therefore un-labelling them as a failure. This may be particularly important to reduce feelings of shame, prevent a decrease or total loss of motivation to quit and promote a more flexible approach to smoking cessation in both patients and clinical staff. Experts agreed and recommended a relapse prevention plan for each patient, addressing personal challenges and risk factors for “slips” and relapse. External and internal triggers such as friends/relatives smoking, alcohol consumption, stress and exacerbation of psychiatric symptoms, can be risk factors for relapse. These should be discussed with the patient and used as a starting point to formulate “emergency measures,” i.e., (preventive) actions to be undertaken in case of confrontation with these triggers. Finding new ways to deal with stress and replacing smoking with other stress-relieving activities is especially relevant in the light of emotion dysregulation, depression and potentially decreased tolerance to stress associated with severe mental illness.

#### Pharmacological Treatment

Participants agreed that medication should be proactively offered to patients in the initial phase to increase chances for successful quitting. Current international guidelines for pharmacological treatment for smoking cessation recommend nicotine replacement therapy (NRT), Varenicline and Bupropion (31). In line with these guidelines, all participants preferred Varenicline and NRT related to their higher effectiveness and fewer side effects. Regarding NRT, there was agreement about not including mouth spray and inhalators for administering nicotine fast through the mucous membranes and hence potential dependency. Participants did not recommend Bupropion as a first-choice medication because of more side effects and interactions with certain anti-depressants and anti-psychotic medication (e.g., Clozapine, Aripiprazole, Risperidone).

Nevertheless, Bupropion could be an alternative in case patients present intolerance or contra-indications for the use of Varenicline (i.e., severe kidney disease or dysfunction) and when patients suffer from attention-deficit/hyperactivity disorder. To the best of the panel's knowledge and clinical experience there is no clear evidence that Varenicline substantially exacerbates present or induces new psychiatric symptoms. One participant referred to a series of trials that examined the safety and effectiveness of these medications for people with SMI (23, 24, 33).

A psychiatrist with comprehensive knowledge of psychopharmaca and smoking cessation medication needs to supervise medication use. The panel also emphasised the importance of recognising that smoking interferes with the metabolism of some antipsychotic medication by enzymes in liver cells. Through this interference, smokers need higher doses of antipsychotic medication. Hence, after smoking cessation, plasma levels need to be determined and medication dose should be adjusted accordingly to avoid strong side effects or unnecessary high levels of antipsychotic medication. The prospect to potentially reduce medication doses was regarded as an important motivating factor for patients. Lastly, there was consensus about the importance of psycho-education about supportive medication so as to build up trust and willingness to use medication. According to the experts, there seems to be some reluctance towards medication for smoking cessation because of expected side effects.

#### Peer Support

There was consensus about the relevance of peer support groups, taking place at least once a week. Most importantly, it offers a safe space to exchange experiences. The participants pointed out the motivational role that a peer group can have when quitting to smoke. The panel considered it essential that the expert-by-experience supporting these group meetings had personal experience with mental illness and addiction in the past and should take a facilitating rather than a leading role. The expert-by-experience has the ability to share their own storey with some emotional distance and make room for patients' experiences with an accepting and hopeful attitude. Topics during these meetings should be determined by the patients themselves, based on their actual experiences while participating in the smoking cessation program.

The involvement of family members was proposed as an optional form of support. Central points for attention are the establishment of rules about smoking in the proximity of the patient and the reduction of other triggers, such as smoking equipment at home (e.g., rolling paper, ashtray). Systemic support by family and/or friends can aid to mitigate these environmental triggers. Similarly, including family members or friends when making the relapse prevention plan can increase chances of quitting success by, for instance, appointing a person who can be contacted in challenging moments of craving. Table 1 outlines the finalized intervention concept with core elements, frequency and duration of the treatment components.

**Table 1:** Final smoking cessation intervention concept.

**Component**	**Core elements**	**Frequency/duration/ dose**	**Responsible mental health care professional**
**Behavioural Counselling**	Group meetings by default, with additional individual counselling if needed. •Motivational preparation for smoking cessation •Psycho-education on: 1. Basic mechanisms of nicotine addiction 2. Physical/mental/emotional effects of smoking cessation in the context of mental health problems 3. Effects of smoking cessation medication •Normalisation of relapse •Personalised relapse prevention plan •Critical assessment of risks and subjective benefits of smoking •Challenging core beliefs and thoughts that maintain tobacco use (including cannabis use if applicable) through CBT techniques such as behavioural experiments •Improving emotion regulation, e.g., dealing with stress •Dealing with withdrawal symptoms and craving	•Month 1–3: weekly •Month 4–12: monthly	Mental health care specialist nurse psychologist
Pharmacological treatment (options)*	(1) Nicotine replacement therapy (chewing gum, patches, pastilles)	Total duration: up to 6 weeks	Mental health care specialist nurse psychiatrist/physician
	(2) Varenicline	Total duration: 12 weeks quit date between week 1 and week 2 of treatment cycle	
	(3) Bupropion	Total duration: 9 weeks	
**Peer support**	•Regular group meetings with non-therapeutic approach •Connecting participants and creating group cohesion •Creating a safe environment in which participants can share experience •Participants can gain hope from positive attitude and deep understanding of expert-by-experience Group meetings do not have fixed content. Participants decide on discussion topics or activities together.	Month 1–3: weekly Month 4–7: bi-weekly Month 8–12: monthly	Expert-by-experience

*Building up and tapering off doses are determined through shared decision making between patient and mental health care professional following national guidelines.

### Compensatory Behaviours, Co-addictions and Harm Reduction

#### E-Cigarettes

According to the panel, e-cigarettes have increasingly become an alternative way of nicotine intake. Advantages of e-cigarette use are their potential to reduce harm of combustible cigarettes, and the possibility of easily lowering nicotine dosages. However, e-cigarette use maintains the habit of smoking and oral fixation, which were described as serious threats to permanent quitting success. Additionally, e-cigarette use can lead to possible long-term negative health effects. Therefore, the panel reached consensus on the fact that e-cigarettes should not be actively promoted. E-cigarettes were, however, proposed as a last resort for patients unresponsive to any treatment offered (i.e., 7–8 unsuccessful quit attempts).

#### Cannabis and Other Substance Use

Cannabis use and the prevalence of cannabis use disorder is high among people with severe mental illness. It can both relieve and trigger psychiatric symptoms, for instance, psychosis. There was agreement that cannabis use has to be treated simultaneously within this intervention since it is often consumed together with tobacco. Therefore, smoking cannabis has the potential to maintain tobacco dependence at the same time. More importantly, cannabis use is discouraged in consideration of its main compound tetrahydrocannabinol (THC), which has a strong psychoactive effect. Positive symptoms such as paranoid ideations, hallucinations and cognitive tendencies contributing to delusions and anxiety can be reinforced by THC. The panel acknowledged a potential subjective beneficial effect of cannabis (e.g., with sleeping problems, low mood or pain relief). If cannabis is indispensable for the patient, the aim will be to find alternative ways of consumption, such as eating or vaporising, rather than quitting its use. Attention needs to be paid to the possibility that, as a compensatory behaviour, the use of other substances may exacerbate or, through disinhibition, contribute to relapse in smoking.

### Implementation

#### Smoking Culture in Mental Health Care and Professionals' Attitude

Firstly, mental health care professionals' perception of and attitude towards smoking is decisive to the intervention's success. Participants reported treatment pessimism among clinical staff regarding the opportunities for smoking cessation of their patients. The panel supposed that pessimistic attitudes of staff about treatment success are related to increased relapse in this specific population. Such a pessimistic attitude can potentially be transferred—implicitly through negligence and lack of support and explicitly through verbal expression of frustration or discouragement—to the patient. Additionally, tobacco addiction is often not included in the primary diagnosis by mental health care professionals. Such diagnostic omission can be an obstacle to offering a structured therapeutic trajectory for smoking cessation and hinder reimbursement for treatment costs from health insurances. Furthermore, mental health care professionals' smoking behaviour is crucial for their motivation to address tobacco addiction with their patients and is also conditional for being a positive role model. Consequently, the panel agreed to select clinical teams for the RCT based on their mind-set and determination about smoking cessation. Two clinical staff members should be appointed based on their motivation, and trained to be responsible for recruiting patients and delivering the smoking cessation intervention. While striving to tailor the intervention as much as possible to the patient's individual needs and personal circumstances, feasibility of its integration into daily clinical routine for clinical staff has to be considered carefully.

## Discussion

The present Delphi study aimed to establish expert consensus on the development and implementation of a smoking cessation intervention for people with severe mental illness, treated in outpatient clinical settings in the Netherlands. To achieve this, we conducted a three-phase Delphi study in which five experts critically reviewed the progressing intervention concept and responded to a number of critical open-ended questions. The panel reached consensus on the intervention's core components [behavioural counselling, pharmacological treatment (NRT, Varenicline, Bupropion) and peer support], their specific content, structure and strategies for optimal implementation. This outcome is in line with recent scientific research findings that showed the safety and effectiveness of these components compared to usual care in reducing smoking and nicotine dependence (27). Studies examining the risk for neuropsychiatric adverse events of smoking cessation medications have not found a significantly increased risk for depression, anxiety, suicidal ideation or suicidal behaviour in people with psychotic and mood disorders (23, 33, 34). These studies also suggest a superior effect of Varenicline compared to Bupropion, NRT and no medication. Psychiatric contra-indications for Bupropion include a diagnoses of bipolar disorder or eating disorders as Bupropion may increase symptoms of depression and/or anxiety and reduce appetite (23, 35), which is in line with the panel's recommendations.

The results of this study help to further specify the contents and structure of these components as well as their contextualisation into current Dutch mental health care. In addition, peer support will make up a fixed part, which has not been standardised in any other study on smoking cessation for people with SMI so far. Mixed-methods studies on peer support groups for people with schizophrenia or psychotic disorders show beneficial effects by improving patients' social networks (36, 37). Therefore, introducing peer support on a regular basis could aid to empower patients during smoking cessation.

Additionally, compensatory behaviours, co-addictions, harm reduction and considerations for optimal implementation were addressed. Despite differences in opinion, the panel reached agreement about the role of e-cigarettes, i.e., being a “last resort” for treatment-resistant patients regarding their smoking behaviour. Two of the experts proposed e-cigarettes a “last resort” to reduce harm of combustible cigarette smoking, while the other three experts did not support the use within clinical practise at all because of habit maintenance and negative health consequences. These discrepancies resonate with current national guidelines on the one hand, that clearly advise against e-cigarette use because of lacking evidence for their safety and the argument that their use could deter long-term cessation and normalise smoking (38, 39). On the other hand, there is research that shows that e-cigarettes are associated with recent quit attempts in people with SMI indicating an interest and potentiality to use e-cigarettes as a quitting aid in this population by reducing smoking of combustible cigarettes (40). One could also argue that through the use of e-cigarettes antipsychotic medication doses can be lowered, as it is the non-nicotinic ingredients of combustible cigarettes that impact enzyme levels and lead to a higher required medication dose (41). In practise, it is a joint process of clinician and patient to negotiate among treatment goals, options and priorities.

Different opinions also arose about whether or not to propose alternative ways of cannabis use. There was, however, clear discouragement of cannabis use in this patient group due to its psychoactive effect and therefore its potential to exacerbate psychotic symptoms. Recent research suggests that cannabis use is associated with a lower likelihood for tobacco abstinence, including those who use cannabis for medical reasons (e.g., pain, insomnia) (42). Hence, these results favour an integrative treatment addressing co-addictions in case of dual use. The research team agreed that patients with alcohol use disorder (AUD) will not be considered for inclusion as AUD could negatively interfere with participation and commitment to the present smoking cessation intervention. Binge drinking and heavy drinking during smoking cessation treatment are associated with a greater risk of smoking lapse (43, 44). An impaired response inhibition, and hence a lower threshold to give in to craving, resulting from alcohol use might account for this greater risk (45, 46). Additionally, alcohol use can increase levels of cigarette craving (47), and cigarette craving is a predictor of smoking relapse (48). Another challenge during the treatment of individuals with AUD is the high rate of treatment dropout (49–51). In conclusion, the treatment of tobacco dependence in individuals with AUD comes with specific challenges that are outside the scope of this intervention and should be tackled in a specially designed treatment.

All aspects considered, consensus on many aspects of the development and implementation of a smoking cessation program in people with SMI treated in outpatients clinical setting was reached. Yet, implementation in realistic clinical settings might still hold unexpected challenges, which will be assessed in a planned RCT subsequent to this study.

Our study has several strengths. Firstly, participants were highly experienced and specialised in treating mental disorders and comorbid addiction or smoking. Secondly, the semi-structured online sessions gave sufficient direction to gather the knowledge needed for the design of the intervention while also allowing new content to emerge. Thirdly, the results portray the complex interplay of physical, psychological, social and environmental factors. Through this, they can endorse a holistic approach to treatment within mental health care institutions and improve the quality of personalised care.

There are limitations to our study. Firstly, our sample size (*n* = 5) is relatively small, which could potentially lower the generalisability of the outcomes. However, for the purpose of our study we selected a small but highly specialised group of experts that we considered sufficient based on relevant knowledge. Despite the small sample size, we do have a broad representation of people with diverse expertise and experiences. Guidelines on the Delphi methodology in scientific research emphasise the selection criteria of having specialised expertise on the subject at hand, rather than suggesting researchers to include a specific number (29, 52). Additionally, there is already existing general consensus on the effective treatment components for smoking cessation. For specifying the contents of these components, an in-depth qualitative study with a smaller number of experts may be more suitable to yield data that can be translated into an intervention protocol. Secondly, even though we included an expert-by-experience to integrate the perspectives from a former patient, we did not include a person who is currently in psychological treatment and is also a current smoker. The inclusion of the broad range of patients' perspectives, which could have added unique content to the design and implementation of the intervention, may therefore be insufficient. To compensate for this to some extent, we encouraged participants to integrate their knowledge and theory of mind about patients' perspectives into their responses. Furthermore, a higher degree of heterogeneity regarding the cultural background of the participants could have increased the intervention's sensitivity for cultural differences in the present patient group.

Overall, this study provides insight into expert opinions on the most relevant elements of the core components and implementation of a smoking cessation intervention for people with SMI treated by FACT teams in the Netherlands. Future research applying the Delphi method for the design of therapeutic interventions should ensure the inclusion of patients in the panel.

## Data Availability Statement

The original contributions presented in the study are included in the article/Supplementary Material, further inquiries can be directed to the corresponding author/s.

## Author Contributions

MK drafted the intervention concept, prepared the Delphi procedure, and drafted the manuscript. TH, JV, MA, and BM revised it. MK and LJ processed all received responses, transcribed the audio recordings, and independently conducted the analysis. BM moderated the panel sessions while. MA moderated two individual sessions. JV contributed intellectually to the final design of the intervention. All authors have read and approved the final manuscript.

## Funding

This work was supported by *Stichting tot Steun VCVGZ* and the Dutch *Ministry of Health, Welfare and Sport*s (Grant Number 258): KISMET—a smoking cessation intervention for people with severe mental illness.

## Conflict of Interest

The authors declare that the research was conducted in the absence of any commercial or financial relationships that could be construed as a potential conflict of interest.

## Publisher's Note

All claims expressed in this article are solely those of the authors and do not necessarily represent those of their affiliated organizations, or those of the publisher, the editors and the reviewers. Any product that may be evaluated in this article, or claim that may be made by its manufacturer, is not guaranteed or endorsed by the publisher.
