# Supplementary material for: Developing a Smoking Cessation Intervention for People With Severe Mental Illness Treated by Flexible Assertive Community Treatment Teams in the Netherlands: A Delphi Study
Source: Front Psychiatry. 2022 Jul 6;13:866779. doi: 10.3389/fpsyt.2022.866779 (PMC9301140; doi:10.3389/fpsyt.2022.866779)
Supplement: Supplementary file 1 [file Table_1.docx]

|  | Questions |
| --- | --- |
| Before round 1 | - How feasible is it to execute the entire intervention in practice? - What are potential challenges related to the implementation of the intervention in practice? - What are your views on using medication for smoking cessation?   - Are there any factors that facilitate or impede the use of medication for smoking cessation? - To what extent do the intensity and frequency of the intervention align with current treatments given by FACT-teams? - What would be the influence of the current COVID-19 pandemic on the practical implementation of the intervention? - With regard to the three components Cognitive behavioural therapy, Medication and Peer support: to what extent do these components align with each other, and to what extent are they complementary?   - What is the best way to implement these components in the intervention? - How can this intervention be incorporated into an already existing lifestyle intervention, in order to increase effectivity? - What are the potential financial barriers?   - For example, are there any financial barriers related to nicotine replacement therapy? - Do you have any other remarks? |
| Before round 2 | **E-cigarettes**   - What are the pros and cons of using E-cigarettes?   - Which pros and cons are most important when deciding to use E-cigarettes in this intervention, in order to increase participants’ wellbeing? - How would you estimate the likelihood of smoking cessation with and without the use of E-cigarettes? - How does the habit of smoking E-cigarettes contribute to the addiction of smoking normal cigarettes? - How should we handle E-cigarette use?   **Comorbid cannabis addiction**   - How should our intervention handle a comorbid cannabis addiction?   **Relapse and relapse prevention**   - What are the crucial predictors of relapse (e.g. self-efficacy, previous failed attempts)? - How can these predictors be used to minimalize the risk of relapse, while simultaneously normalizing relapse?   **Experts by experience, peer support and involvement of family members**   - How is peer support currently used in treatment for smoking cessation? - What are the effective components of peer support groups, and which meeting frequency would be considered ideal? - How can family/partners/friends be involved in the intervention?   **Duration**   - How feasible is it for FACT-team employees to plan weekly counselling sessions?   - What is the ideal hours per week for counselling sessions?   - What is the ideal total number of counselling sessions?   - Should counselling sessions be delivered as individual or group sessions?   **Implementation within FACT-teams**   - How high would you estimate the percentage of people with an EPA that are willing to stop smoking? - Regarding FACT-teams’ workload: what is the maximum amount of patients that one FACT-team can include and treat in the intervention? - How many FACT-teams are needed to include approximately 318 patients? |
| Before round 3 | **Pharmacotherapy**   - How much supervision by a doctor/psychiatrist/other specialist is needed, and how much supervision is feasible?   **Behavioural support**   - How many weekly/monthly individual sessions are needed in the first intensive intervention period? - What is the ideal ratio of individual support and group sessions?   **Peer support and experts by experience**   - What is the average availability of experts by experience in (certified) FACT-teams?   **Relapse and relapse prevention**   - Which skills should be included in the training for FACT-teams, regarding relapse and relapse prevention?   **E-cigarettes**   - How can we standardize the decision making process of using E-cigarettes in the intervention?   - What amount of quit attempts should we consider as ‘sufficient’?   **Comorbid cannabis addiction**   - Should we exclude participants with a diagnosis of ‘Cannabis use disorder mild/moderate/severe’ according to DSM-5?   - Why/ why not?   **Interaction with antipsychotic medication**   - How often should participants meet with a psychiatrist/doctor?   **Practical implementation within FACT-teams**   - How many FACT-teams are needed in order to recruit approximately 300 participants? - Are FACT-teams qualified to execute the intervention?   - Which aspects are most important during the training for FACT-teams? - Do you think that FACT-teams are willing to offer additional support via phone calls? - Do you think that FACT-teams have enough time to offer these additional phone calls? |
